# Synaptic Biomarkers in Alzheimer's Dementia: A Meta‐Analysis

**DOI:** 10.1002/alz70856_105493

**Published:** 2026-01-07

**Authors:** Amish Gaur, Jinghan Jenny Chen, Melissa Wong, Yejin Kang, Danielle Tahoulas, Damien Gallagher, Nathan Herrmann, Krista L Lanctôt

**Affiliations:** ^1^ University of Ottawa, Ottawa, ON, Canada; ^2^ Sunnybrook Research Institute, Toronto, ON, Canada; ^3^ University of Toronto, Toronto, ON, Canada; ^4^ Department of Psychiatry, Temerty Faculty of Medicine, University of Toronto, Toronto, ON, Canada

## Abstract

**Background:**

Alzheimer's dementia (AD) is a neurodegenerative condition characterized by progressive cognitive decline. Synaptopathy—defined as loss and dysfunction of existing synapses—is a hallmark pathological feature of AD and can directly contribute to underlying cognitive deficits. In this study, we meta‐analyzed several cerebrospinal fluid (CSF) and blood exosomal biomarkers associated with synaptopathy in AD and healthy controls (HCs).

**Method:**

Original peer‐reviewed articles that reported synaptic biomarker concentrations in CSF or blood exosomes were reviewed. Specifically, synaptosome associated protein‐25 (SNAP‐25), growth associated protein‐43 (GAP‐43), neuronal pentraxin receptor (NPTXR), neuronal pentraxin‐1 (NPTX‐1), neuronal pentraxin‐2 (NPTX‐2), complexin‐2, syntaxin‐1B, syntaxin‐7, and vesicle‐associated membrane protein‐2 (VAMP‐2) in AD and HCs were included for meta‐analysis. A random‐effects model was used to determine standardized mean differences (SMDs) and 95% confidence intervals (CIs). Heterogeneity was quantified using I^2^.

**Result:**

The meta‐analysis included 43 study cohorts. In CSF, concentrations of SNAP‐25 (N_AD_/N_HC_ = 394/539, SMD [95% CI] = 1.08 [0.73, 1.42], *p* < 0.001; I^2^ = 81.43%), GAP‐43 (N_AD_/N_HC_ = 851/557, SMD [95% C] = 1.02 [0.69, 1.34], *p* < 0.001; I^2^ = 83.97%), and VAMP‐2 (N_AD_/N_HC_ = 398/490, SMD [95% CI] = 0.32 [0.05, 0.60], *p* = 0.02; I^2^ = 64.91%) were elevated, and NPTXR (N_AD_/N_HC_ = 575/470, SMD [95% CI] = ‐0.68 [‐0.96, ‐0.40], *p* < 0.001; I^2^ = 75.59%), NPTX‐1 (N_AD_/N_HC_ = 344/333, SMD [95% CI] = ‐0.48 [‐0.64, ‐0.31], *p* < 0.001; I^2^ = 9.05%), and NPTX‐2 (N_AD_/N_HC_ = 462/496, SMD [95% CI] = ‐0.78 [‐1.02, ‐0.54], *p* < 0.001; I^2^ = 66.43%) were decreased in AD compared to HCs. In blood exosomes, SNAP‐25 (N_AD_/N_HC_ = 161/159, SMD [95% CI] = ‐1.05 [‐1.28, ‐0.82], *p* < 0.001; I^2^ = 0%) and GAP‐43 (N_AD_/N_HC_ = 110/110, SMD [95% CI] = ‐1.66 [‐2.43, ‐0.88], *p* < 0.001; I^2^ = 77%) concentrations were decreased in AD.

**Conclusion:**

This study found that several synaptic biomarkers were significantly altered in AD in CSF and blood exosomes. There was significant heterogeneity for most comparisons (with the exception of CSF NPTX‐1 and blood exosome SNAP‐25) that remains to be explored. Nonetheless, further review of the identified biomarkers may provide fundamental insight into AD pathophysiology and disease trajectory.

